# Staging of diabetic tendinopathy in the calcaneal tendon of Wistar rats

**DOI:** 10.1186/1758-5996-7-S1-A8

**Published:** 2015-11-11

**Authors:** Ana Camila Nobre de Lacerda Brito, Márcio Almeida Bezerra, Cybelle da Silva Nery, Marina Lira Cavalcante, Camilla Rodrigues de Souza Silva, Rodrigo Santos Walter, Marina Cerqueira Rosdaibida Gomes, Laylla Marjorye Rebouças Bezerra, Magno Felipe Holanda Barboza Inácio Teixeira, Silvia Regina Arruda de Maraes

**Affiliations:** 1 Universidade Federal de Pernambuco, Recife, Brazil

## Background

Diabetes Mellitus (DM) causes biomechanical tendon fragility and it may evolve to degeneration and rupture[1]. Although it has been demonstrated the presence of associations between periarticular and musculoskeletal injuries with diabetes[2,3], it has not yet been elucidated from which moment these changes are installed in tendon structure.

## Objective

To identify the beginning of the biomechanical changes of diabetic tendinopathy, through the study of staging in the calcaneal tendon of Wistar rats.

## Materials and methods

In the 100 days of life, 16 male Wistar rats were induced to DM by intraperitoneal administration of Streptozotocin and divided into four groups according to the number of weeks post induction in which the tendons were collected: Group 1st week post induction (1st WEEK PI), Group 2nd week post induction (2nd WEEK PI), Group 3rd week post induction (3rd WEEK PI) and Group 4th week post induction (4th WEEK PI). After the trial period, the animals were anesthetized and the left calcaneus tendons were removed for the mechanical traction rehearsal. From the rehearsal the following parameters were evaluated: Elastic Modulus (MPa), Maximum Tension (MPa), Specific Strain (%), Maximum Strength (N), Cross-sectional Area (mm2). For statistical analysis it had been used the ANOVA and Tukey post hoc.

## Results

In the 16 animals induced, two have not become diabetic (one rat of the 3rd WEEK PI and one of the 4th WEEK PI). Maximum strength and maximum tension did not differ between the groups. The cross-sectional area and the specific strain showed reduction in 3rd WEEK PI (p=0.012; p=0.006, respectively) and in the 4th WEEK PI (p=0.005; 0.008, respectively) compared to the 1st WEEK PI; specific strain decreased also in the 3rd WEEK PI (p=0.002) and 4th WEEK PI (p=0.002) compared to 2nd WEEK PI. On the other hand, the elastic modulus was greater in the 3rd WEEK PI (p=0.047) and 4th WEEK PI (p=0.032) compared to 1st WEEK PI, besides the increase in the 4th WEEK PI (p=0.036) compared to 2nd WEEK PI.

## Conclusion

The study of staging the calcaneal tendon of rats induced to experimental diabetes identified biomechanical and structural changes of calcaneal tendon from the 3rd week post induction, suggesting that from this phase the tendons become more susceptible to degeneration and rupture when subjected to tension.
